# Techniques of disinformation: Constructing and communicating “soft facts” after terrorism

**DOI:** 10.1111/1468-4446.12735

**Published:** 2020-01-31

**Authors:** Martin Innes

**Affiliations:** ^1^ Crime and Security Research Institute Cardiff University Cardiff United Kingdom

**Keywords:** disinformation, social media, terrorism

## Abstract

Informed by social media data collected following four terror attacks in the UK in 2017, this article delineates a series of “techniques of disinformation” used by different actors to try and influence how the events were publicly defined and understood. By studying the causes and consequences of misleading information following terror attacks, the article contributes empirically to the neglected topic of social reactions to terrorism. It also advances scholarship on the workings of disinforming communications, by focusing on a domain other than political elections, which has been the empirical focus for most studies of disinformation to date. Theoretically, the analysis is framed by drawing an analogy with Gresham Sykes and David Matza's (1957) account of the role of “techniques of neutralization” originally published in the American Sociological Review. The connection being that where they studied deviant behaviour, a similar analytic lens can usefully be applied to disinformation cast as “deviant” information.

## 1 INTRODUCTION

Disinformation can be defined as “deviant information.” For where information is imparted to enhance awareness, insight, and understanding, disinforming communications blend intent and action to distort, deceive, and dissemble. Framed in this way, the causes and consequences of disinformation have recently risen up the hierarchy of public and political concerns. Deliberately misleading public communications have been found embedded within a number of information, interference, and influence operations and campaigns, authored and amplified by a range of state and non‐state actors, variously targeting: significant democratic events, such as the 2016 US presidential election; “anti‐vaxxer” narratives, that have significantly reduced “herd immunity” for highly infectious diseases such as measles; fueling inter‐ethnic conflicts in geo‐political “hot‐spots” such as Syria; and climate change denial conspiracies (see Kakutani, 2018; Pomerantsev, 2019). Disinformation is thus both a problem in and of its own right, but also because of how it is functioning as a centrifugal influence upon other pressing social problems. This article attends specifically to the aftermath of terror attacks, using this focus to illuminate some broader and deeper patterns in how disinforming messages are constructed and communicated.

Conceptually, the analysis draws inspiration from Gresham Sykes and David Matza's (1957) influential article published 50 years ago now, where they delineated five “techniques of neutralization” used to mitigate and assuage any sense of guilt experienced when engaging in deviant behavior. According to Downes and Rock (2007), these techniques imprint honorable motivations onto dishonorable acts, albeit this may twist and manipulate empirical reality. They are routinely invoked nonetheless, to preserve self and social identity in the face of public information that might otherwise tarnish or discredit an individual's status or reputation. But where Sykes and Matza were concerned with deviant behavior, herein the focus is upon deviant information.

Processes of self‐ and social identity fabrication are currently undergoing radical revision (Marwick, 2013), driven by interactional and institutional reconfigurations associated with the information age (Castells, 1997). Amoore and Piotukh (2015) assert that data‐processing algorithms are increasingly influential “instruments of perception,” framing the social issues that are collectively attended to, and which are neglected. This ability to steer the constructing of “public problems” is pivotal to how social communications platforms and their data are impacting upon the ordering of social and political realities (Couldry & Hepp, 2017; Gonzalez‐Bailon, 2017).

While such developments have been widely eulogized for their democratizing and progressive potential (Brym, Godbout, Hoffbauer, Menard, & Zhang, 2014), more recently a “darker” side has impinged upon discussions of digital communications and their consequences. Political and public debate has increasingly centered on how new social media technologies are affording the routine communication of misinformation and disinformation (Kavanagh & Rich, 2018; Kakutani, 2018). These social processes are revivifying some long‐standing interests of social science but with a contemporary twist, such as has happened, for example, with the introduction of concepts of “computational propaganda” (Woolley & Howard, 2019) and “network propaganda” (Benkler, Faris, & Roberts, 2018).

There is, after all, a long‐standing and well‐developed literature on the causes and consequences of rumors as “improvised news” (Shibutani, 1966), as well as the social psychological motivations undergirding how individuals construct and communicate them (Allport & Postman, 1947; Fine, Campion‐Vincent, & Heath, 2005). Likewise, conspiracy theories have been widely studied (Douglas, Sutton, Jolley, & Wood, 2015; Goertzel, 1994), as has mass media's propagation of factually incorrect stories and narratives—in contemporary parlance, “fake news.” What is more innovative, is how the contemporary media ecosystem has amplified the impacts and reach of such communications (Couldry & Hepp, 2017). There is empirical evidence that lies and untruths online typically “travel” further and faster than do verified “facts” (Vosoughi, Roy, & Aral, 2018).

Particular attention in the current moment has pivoted around how a stream of rumors, propaganda, conspiracy theories, and “faked news” are degrading public trust in institutions in general, and the processes of government and democracy in particular (Gonzalez‐Bailon, 2017; Greenhill & Oppenheim, 2017; Kavanagh & Rich, 2018; Oh, Agrawal, & Rao, 2013). However, deploying rumors and conspiracy theories to modify how people think, feel, or behave has not been confined to democratic processes. By studying these other situations, and bringing any findings alongside those derived from analyses of political communication, there is potentially much to be learnt about how digital disinformation is designed and delivered. Informed by empirical data systematically collected by monitoring social media following four terror attacks that took place in the United Kingdom in 2017, the analysis detected a number of distinct “episodes” where false and misleading definitions of the situation were communicated. Conceptually, these are used to model a series of “techniques of disinformation,” that in several important respects, resonate with the empirical and theoretical insights foregrounded by Sykes and Matza half a century ago.

Set against this backdrop, three principal claims are proposed. First, the analysis enriches understanding of the aftermaths of terrorist violence. For although considerable effort has been directed towards the study of terrorism over the past two decades, somewhat surprisingly, social reactions to terror events have remained relatively neglected. Far more work has gravitated around radicalization and the acquisition of terrorist motivations, than with empirical investigations of what happens when plots cannot be interdicted (English, 2016).

The second claim relates to the value of studying post‐attack situations as conducive settings for the communication of misleading and false information. A significant body of crisis communications research documents how, following dramatic and unexpected emergencies, rumors, and conspiracies abound (Seeger & Sellnow, 2016). Moments of crisis generate collective uncertainty about what precisely is transpiring and how it should be interpreted, thus rendering participants and audiences highly “influence‐able.” The particular contribution the present article makes is surfacing how, in the contemporary media ecosystem, the impacts of crisis events are routinely shaped by multiple, interspersed, and interacting instances of inaccurate information. This is significant inasmuch as the most influential studies of rumors, conspiracy theories, and fake news, all share a tendency to isolate and focus upon a particular instance and study it intensively. Contrasting with which, herein it is suggested that a swirling mix of claims and counterclaims, have to be accounted for, if we are to understand how and why different “soft facts” gain traction.

“Soft fact” captures how some knowledge claims are plastic, malleable, and contested (Innes, 2014). Where the more familiar notion of a “hard fact” is attributed objectivity and stability, a soft fact is frequently and sometimes repeatedly manipulated and edited, albeit it is afforded contingent authority and credibility by some. In this article, soft fact is deployed as a “master concept” encompassing rumors, conspiracy theories, fake news, and propaganda. As such, it covers both misinformation (inadvertently misleading communications) and disinformation (a deliberate attempt to deceive). The advantage of this approach is in drawing out common patterns in how and why specific inaccurate communications arise, and how they overlap and intertwine in highly charged emotional settings, where verified information about what has happened is lacking.

The third main claim resides in conceptualizing a series of techniques of disinformation. Echoing well‐established precepts of contemporary social theory, Dahlgren (2005) delineates three main analytic levels for scholars of how internet technologies have impacted political communication and democratic processes: the structural, representational, and interactional. The greatest volume of work pivots around the application of network analysis methodologies and quantitative data, mapping the nodes, and links structuring the dissemination of disinformation (Centola, 2018; Gonzalez‐Bailon, 2017). A second grouping of studies displays traits analogous with Dahlgren's representational dimension, concerned with how certain visual and linguistic “grammars” configure the information and meanings communicated. Also included here are “sentiment analyses” (Salganik, 2017). Located between these two levels is a more “interactional” focus, attending to the behaviors used in transmitting and receiving disinforming communications. It is this level of mid‐range theory that the techniques of disinformation align with.

The next section outlines how the empirical data were collected and analyzed. This sets up a discussion of the individual disinformation techniques, designed to illuminate how these methods for communicating false and inaccurate information are organized and work. The article concludes by considering why these methods are able to influence peoples' perceptions and beliefs so readily.

## 2 RESEARCH DESIGN AND METHOD

Data were collected in the wake of four terrorist attacks that took place in the UK in 2017. The first attack on Westminster Bridge (March 22, 2017), near the Houses of Parliament, was committed by a lone offender driving a van into pedestrians, before fatally stabbing a police officer. This attack methodology was very different from the Manchester bombing of the Ariana Grande concert (May 22, 2017), which was far more sophisticated in its planning and preparation. The Manchester attack was followed on June 3, 2017, back in London, by a marauding attack by three individuals, again using vans and knives. The fourth attack occurred on June 19, 2017 involving a single perpetrator with extreme far‐right motives, targeting Muslim worshippers in Finsbury Park.

A total of just over 30 million data points were collated from across multiple social media platforms, with a particular focus upon Twitter, utilizing the Sentinel platform. Sentinel comprises a suite of data collection and analysis “apps” and algorithms, with similar collection and processing functionality to many commercial packages (Preece et al., 2018). However, whereas the latter are “black boxed” (Pasquale, 2015), Sentinel is “glass boxed,” enabling researchers to investigate how particular decisions and choices in terms of data collection, processing and analysis, structure and shape resultant data flows. Sentinel's data collection is organized around a series of user configurable “channels,” comprising up to 400 search terms that filter out irrelevant material, while capturing units of social media traffic that, because of their textual content, are likely connected to the subject of interest. This structure enables the system to work within the 1% limit of total traffic volumes freely available through the Twitter “firehose.”

Data reduction to focus upon specific disinforming communications was accomplished by researchers identifying a series of “episodes” that appeared especially interesting and relevant, through contemporaneous “real time” monitoring. These were subsequently developed in “slow time” once the event had concluded. Episodes are defined events within a larger narrative, that can be isolated and studied intensively to draw out wider learning in terms of what happens and why. Clear analogies can be made with the principles of Manning's (2016) “pattern elaborative theory.” He suggests that, engaging an interplay between “exemplary evidence” and key theoretical precepts, can distil regularities and patterns in behavior and conduct not previously recognized or perceived.

A total of 22 “episodes” involving the communication of one or more soft facts across the four attacks were identified for detailed case study analysis. Each episode was subject to qualitative analysis of text and imagery, with a particular accent placed upon digital behaviors and how people “do things to information, to do things with information.” It was thus guided by an analytic interest with how human users interact with each other, with the information they exchange and the technological instruments they engage (Housley et al., 2018). This “digital behavioural analytics” illuminates how disinformation is transferred between actors, and how the blend of content and action guides any influence accomplished. As such, much of the analysis was directed towards understanding the proximate causes and consequences of specific soft facts, and their role in influencing how public understandings, and definitions of the situation, were constructed and contested. Each episode illuminated a particular facet of how soft facts function, that when blended together, enabled more comprehensive insights. It was the observed patterns distilled from this process that informed development of the conceptualizations that are the article's focus.

Contemporary journalistic and political discussions of disinformation have frequently pivoted around evidence of a recent campaign by the Russian state to interfere in the democratic processes of the United States.^1^ As such, part of this article's contribution, as rehearsed in the Introduction, is in examining an alternative policy domain. Namely, the four UK terror attacks. The accounts concerned were attributed a Russian identity on the basis of material released by the US Senate investigation.^2^

According to Webb et al. (2016), the rapid development of social media and its study means that there is not yet a clear consensus about ethical protocols for collecting and reporting these kinds of data. Given the contentious and sensitive nature of many of the issues, for the most part, data herein are reported anonymously. A dimension of the analysis of potential wider import to the ethics of social media research, concerns the scale and prevalence of retrospective editing and deletions of messages and communications that is evidenced. Some academics (Williams, Burnap, & Sloan, 2017) have suggested that ethical practice requires analysts do not reproduce messages that authors have decided to remove. The issue with this approach that the current study highlights is that it could render academic research complicit in the reproduction of disinformation and the editing of the historical record, in that it does not capture the full details of how and why certain events unfolded in the ways they did.

## 3 SOFT FACTS IN THE WAKE OF TERROR

“Huge bang in Manchester 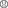.” (22:34:10)

“Just heard a loud bang and now siren + announcement voice going off from Victoria station :/ #Manchester” (22:34:21)

“Wtf is happening in Manchester arena? Massive bang & everyone is running & screaming, people are crying” (22: 35: 18)

In the way that such things happen nowadays, these messages sent within short intervals of each other, by three Twitter users unknown to each other, announced a horrific act of violence had just taken place. Salman Abedi had detonated a bomb killing 22 and injuring over 500 at the Manchester Arena, at around 22:30 on the evening of May 22, 2017. Although with the benefit of hindsight, we now know what these tweets were all referring to, at the moment when they were communicated, the cause of “the bang” was uncertain.

Shortly after these messages, others followed proffering potential explanations. Several attributed it to a “balloon popping.” Others focused instead upon a “speaker exploding.” Of course, these accounts did not sustain credibility for very long as the scale of the injuries suffered became more apparent. However, what these messages do collectively convey is how, in the immediate wake of extreme violence in a moment of high uncertainty, misinformation is commonplace and can be highly influential upon initial public interpretations and sense‐making.

Messages of this type are archetypal soft facts. As evidenced by the online behaviors of other social media users responding to their transmission, they were assigned a “factual” status and epistemic authority because of the credibility attributed to their sources who were “at the scene.” Equally, however, they were “soft” in that this authenticity was contingent and temporary. As they were shown to be false, people refined and revised their understandings. This is coherent with the general tenor of research about what happens in the minutes and hours after a terror attack, where understandings are highly contingent and uncertain (Innes, Roberts, Preece, & Rogers, 2018).

## 4 SEEDING

Looking across the empirical data from the four attacks, in tandem with previous studies, it seems that social media communications immediately following a terror event are often inaccurate and misleading. This is not always intentional, resulting instead from a “media logic” privileging speed of communication over accuracy and validation (Altheide, 2006), thus constituting misinformation rather than disinformation. The key analytic point to be made is how, in “seeding doubt” about what has actually transpired, misinformation creates the conditions for the communication of more deliberately framed disinformation.

During the early reactions to the Manchester attack, there were multiple instances of doubt “seeding.” For instance, soon after the initial reports began circulating, pictures from the scene were being attached to Twitter messages and posts to Facebook. However, several response messages were then sent claiming the images were a hoax, and related to a police training exercise at the Manchester Arena earlier that year:

22:39 “If you see people sharing this image re Manchester it's already been debunked as being from a training exercise video.”

**Figure 1:**
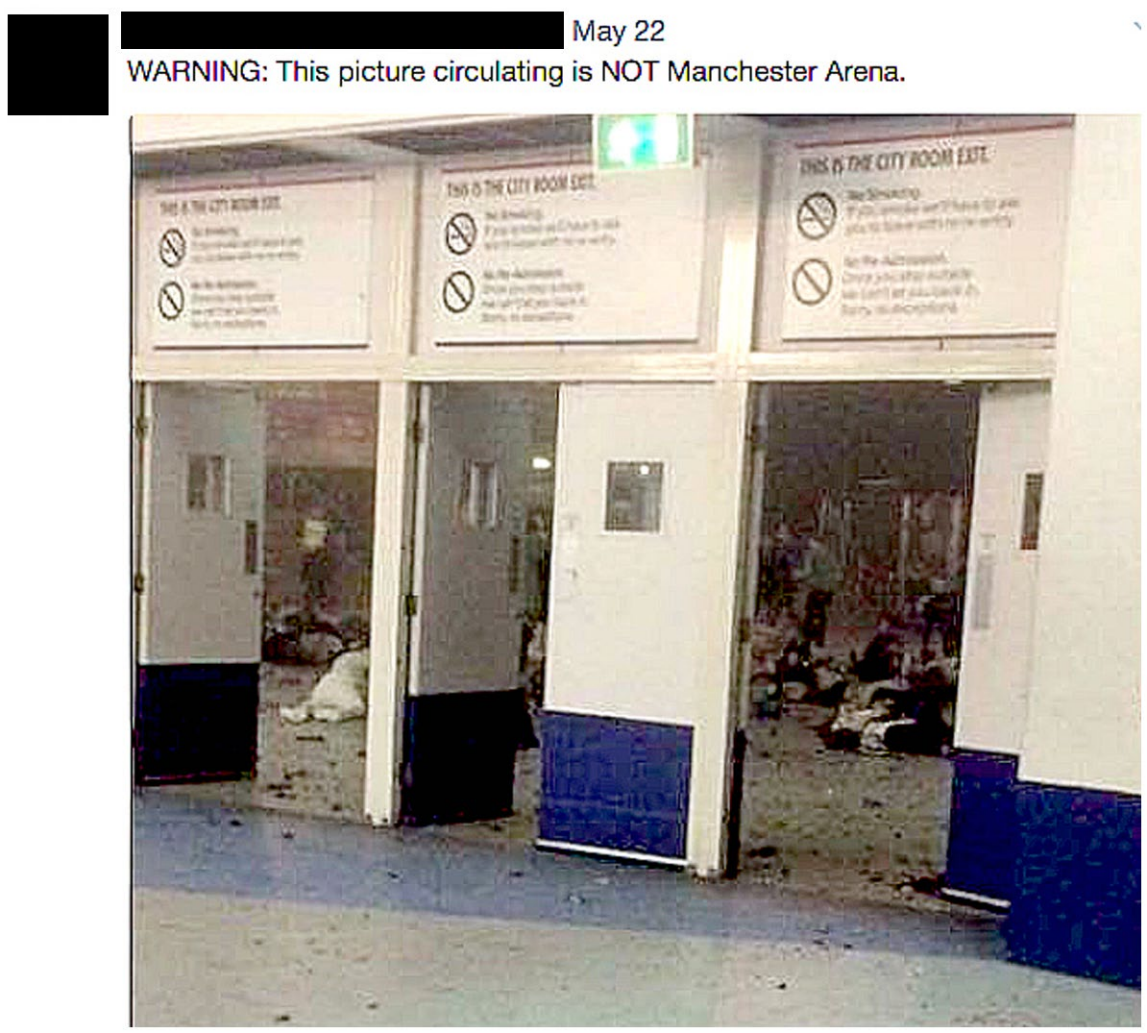
Tweet following Manchester arena attack [Colour figure can be viewed at https://www.wileyonlinelibrary.com]

This message was retweeted approximately 240 times in a rapid pulse of messaging activity. Relatedly, a *New York Observer* columnist posted the image and text in Figure 1, receiving 282 retweets and 364 “likes.” Several other users adopted similar lines doubting an explosion had occurred.

More detailed images from the scene quickly followed, clearly depicting mass casualties. This led many previous “doubters” to revise their positions, but not all did. In the days and weeks following the attack, messages from a relatively small number of users continued claiming that it was an elaborately staged deception. More generally, the use of visual images to try and validate claims to veracity is an important element of digital persuasion (see “truthing” section below).

A second episode further illuminates the blending together of uncertainty and misinformation to create a “space” where “disinforming” communications can acquire traction. It involved the misidentification of “Abu Izadeen” as a suspect for the Westminster Bridge attack.

The rumour about the high profile Islamist extremist Izadeen first appeared via an unverified Twitter account “@News_Executive,” positioned as an online source for fast “breaking” news stories:

BREAKING UPDATE: Reports name the Westminster terrorist suspect as hate preacher Abu Izzadeen (Trevor Brooks) from Clapton in Hackney. (March 22, 2017, 17:59)

This message was disseminated widely and rapidly, receiving 442 “likes” and 1,455 retweets that evening. Subsequent Twitter and Facebook communications used images of the suspect at the scene, alongside a media photo of Izzadeen, to highlight several visual similarities between the individuals concerned. However, approximately 2 and 6 min after @News_Executive, two foreign news outlets—*La Stampa* (Italian) and *Dreuz* (French)—also named Abu Izzadeen as the attacker. Intriguingly, from a disinformation perspective, both articles were modified the following day to claim that Channel 4 News and other British mainstream media were responsible for misidentifying the Westminster terrorist, effectively attributing the “fake news” to other sources. Fourteen minutes after @News_Executive's message, Abu Izzadeen's Wikipedia page was edited to claim he was responsible for the Westminster attack. Wikipedia provides an audit trail for all previous editions and changes, revealing Izzadeen's Wikipedia page was edited 84 times that day.

Channel 4 News commenced their evening television broadcast at 19.00 with the main presenter, on location at Westminster, naming Izzadeen as a suspect. Thirteen minutes later, Rym Momtaz (an ABC producer) tweeted she had contacted Izzadeen's solicitor who had confirmed Izzadeen was still serving a prison sentence for breaching an anti‐terror order and could not have been the attacker. About 20 minutes after Channel 4 News, two UK‐based news‐outlets—*The Independent* and *IBTimes*—published online articles reporting Abu Izzadeen as the attacker. Similarly to *La Stampa* and *Dreuz,* both UK articles were subsequently amended (or deleted). Despite this, their previous stories and approximate time of posting can still be discerned through detailed analysis of Twitter.

About 35 minutes into the one‐hour program, Channel 4 News's Senior Home Affairs Correspondent, Simon Israel, started to voice doubt about the information he had previously provided on air. At 19:54 (the end of the program), the presenter revealed Channel 4 News had been contacted by Izzadeen's brother stating he was still in prison. At about 20:50, both Simon Israel and Ben de Pear (Channel 4 News's editor) tweeted apologies for the mistake on the basis that “this was a fast‐moving story” where conflicting information was coming to light:

Simon Israel: The source I trusted, but ultimately I made a mistake. This time got it wrong. Abu Izzadeen is in prison. (March 22, 2017, 20:50)

Despite this full retraction, the soft fact claiming Izadeen's involvement continued to circulate on social media for several days afterwards. Notably, it was shared and retweeted in high volumes by senior figures in the far‐right Britain First group, and by prominent alt‐right accounts in the United States. In terms of its usage by these other groups, it provided an opportunity to engage in “emulsification” (see below), blending the current crisis event with a wider set of issues they wanted to promote.

Three aspects of this episode are worth foregrounding. First, there is the interaction between social and mainstream media sources in constructing and communicating soft facts. Implicit in many contemporary discussions, has been the idea that the communication of rumors and conspiracies is a particular pathology of social media systems. This case finesses this narrative in important ways. Also significant from a disinformation perspective, is evidence of how several media organizations have “retroactively” amended the histories of what they said or did at the time, attempting to edit out discrediting information from their timelines. They have themselves engaged in “disinforming” behaviors.

More broadly, this case study conveys the “messy,” contingent and complex nature of disinformation as an “artefact,” as it is subject to multiple edits, rewrites, and revisions as it travels through the media ecosystem. The key point about “seeding doubt” as a technique of disinformation is that it is not designed to convince members of the audience to believe a particular interpretation or set of facts. Rather, it works by rendering people into a state where they do not know what to trust and who to believe.

## 5 DENIAL OF CREDIBILITY

In their original five techniques of neutralization, Sykes and Matza (1957) framed three as involving acts of denial (denial of responsibility, injury, and of the victim). Their perspective was an explicit influence upon Cohen's (2005) later, more rigorous, work on the politics of “literal, interpretive, and implicatory” forms of denial. Focused upon the contemporary information environment and the conduct of disinformation communications, a particular formulation of denial can be detected. “Denial of credibility” involves attacking the source of information and is often inflected by analogies to the kinds of technique that were Sykes and Matza's preoccupation.

Examining the empirical social media data tracking reactions to the four attacks, in each case there were a small number of accounts claiming it was a hoax. Typically, these engaged in detailed dissections of particular aspects of the incident to attack the credibility of those involved in the event, and of the media institutions colluding in the conspiracy by reporting it as “real.”

Oftentimes this could be quite grotesque. For instance, following the Westminster Bridge attack, a picture was circulated across multiple social media platforms of a victim's body lying under a red London bus, with the head obscured and legs protruding from beneath the wheels. The force of the collision and weight of the vehicle, made the corpse look akin to a mannequin, with comments such as the following accompanying the picture: “The person is not injured or run over. It's just for shock effect, nothing else, meant to manipulate public consciousness.” This denial of credibility was being used against “the evidence” that other social media users were sharing.

More often, however, attempts to deny credibility were directed towards individuals and/or institutions. Following the Westminster Bridge attack, Mark Rowley (national lead for Counter Terrorism Policing and Acting Deputy Commissioner for the Metropolitan Police), made two statements informing the public about what had happened and developments in the police investigation. In his evening press conference, he stated:

we must recognize now that our Muslim communities will feel anxious at this time given the past behaviour of the extreme right wing and we will continue to work with all community leaders in the coming days.

This comment, highlighting a potential for extreme right‐wing violence, which constituted a small fraction of an otherwise lengthy and informative statement, was significant. It was the first time such a pre‐emptive statement had been made by police in this manner, reflecting learning gleaned from previous terror attacks (Innes, Innes, et al., 2018). It also triggered intense and aggressive negative reactions from far‐right supporters. These responses escalated into the construction and dissemination of a meme, shared and reposted extensively by several high profile far‐right groups and “personalities,” for example Tommy Robinson and the British National Party.^3^ It contained Rowley's image on the left, an extract of his “far‐right concerns” quote on the right, and an alternative “truth claim” at the bottom:

No mention of the concerns of the English community feeling anxious concerning Muslim terrorism and prime example of the liberalism that is killing England.

The clear suggestion being that the police and other “elite” groups in society do not understand the concerns of “ordinary” people. Other variants of this included:

Fuck them and fuck you Mr Rowley! What about us ……… THE BRITS? The people you promised to protect and serve!! RT 0. (March 24, 2017, 9:44 AM)

Albeit, adopting slightly different vectors of attack, collectively such reactions were intended to try and disrupt the authority and credibility of Rowley as a persuasive messenger. In so doing, they utilize several verbal formulations familiar from Sykes and Matza's methods of neutralization, including “denying the victim,” “condemning the condemners,” and “appealing to higher loyalties.”

It is unclear how much impact such attempts to undermine the credibility of authoritative figures/institutions actually achieve. More generally, however, their very presence signals how high intensity and intemperate rhetoric are used to underpin disinformation, and thus shape the tenor and tone of online interactions around contentious issues. It is suspected that such processes suppress alternative narratives and viewpoints being introduced into the digital conversations, as some users do not want to engage in a conflictual situation.

## 6 EVENT GHOSTING

“Event ghosting” involves fabricating, through disinforming social media communications, aspects of an episode that did not really happen. By inserting these “invented” details into a wider narrative to augment it, the meaning is “turned” or changed in some way. It is an important technique of disinformation as these illusory “digital apparitions” can exert considerable influence upon public understandings of the wider episode, even after they have been empirically disproven.

**Figure 2:**
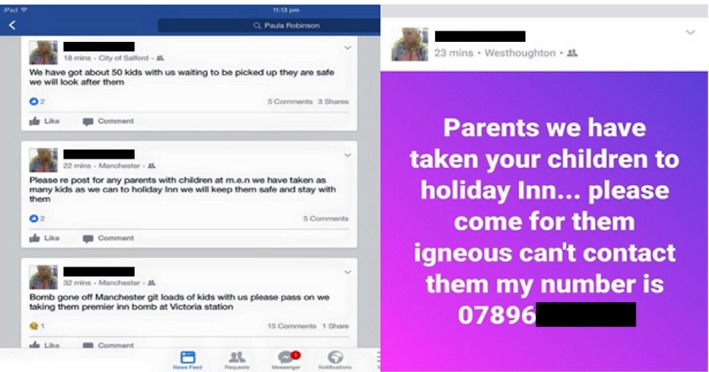
Posts to Facebook [Colour figure can be viewed at https://www.wileyonlinelibrary.com]

One episode following the Manchester attack exemplifies several key features of event ghosting as a mode of communicating soft facts. It concerns the activities of a woman who, after the bombing, claimed to have taken a number of children separated from their parents to a local hotel, where she was keeping them safe (Figure 2). At the time, and for a period afterwards, this story was communicated widely on the main social media channels, including a contact number for worried parents to phone. It was also picked up by multiple press and broadcast media outlets who publicized it further. The main protagonist was a real person and she issued her genuine mobile telephone number on the night of the attack. However, she did not shelter any unaccompanied children in a hotel. It never happened.

While it is difficult to remotely divine the individual's motives and intents, we can though identify the presence of a series of influencing techniques used to construct a persuasive and plausible narrative. At around 00:30AM, the first of four messages sent in quick succession to each other were posted to her Facebook page:

Bomb gone off Manchester git loads with kids with us please pass on we taking them to premier inn bomb at Victoria station. (March 23, 2017, 12:30 AM approximate time)

In the emotionally charged post‐terror environment, these messages generated a huge response via Facebook screenshots being circulated on Twitter. There was a spike in Twitter traffic around this time concerned with missing children. The following message alone was retweeted over 17,600 times:

#Manchester There are over 60 children without guardians at Holiday Inn if you're missing or can't get hold a loved one ring 07896711XXX RT.

This pattern of collective behavior captures some of the “fuzzy” boundaries that exist between misinformation and disinformation in online environments, and hence why adopting a concept such as “soft fact” might be preferable. For what can be observed is how the originator of the message was engaged in disinformation communication; however, the thousands of people that shared and reposted her messages were doing so mistakenly believing it was genuine. Their misinformation significantly amplified the reach of the disinformation. Other case studies reported herein, demonstrate that this coupling can sometimes be reversed, with misinformation creating an environment conducive to disinformation.

One reason for these high levels of online mobilization may be that it helped onlookers online to feel they were making a positive contribution when confronted with an atrocity in Manchester. But also, by placing herself “at the scene” the female protagonist claimed “epistemic authority” and appeared to be engaging in important pro‐social action (looking after lost children) in the absence of any specific official advice. This was supported by her “back story” that positioned her as a mother and grandmother herself.

Within an hour of posting to Facebook, the social media audience were lauding her and calling for her to be rewarded for her actions:

Can we give praise to this lady “[XXXX]” who is looking after missing children, when evil strikes the people hit back 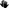 #Manchester. (23/05/17: 12:43AM)

She was labeled the “Angel of Manchester,” an identity the *Daily Mail* newspaper used in an article 7 months later about her being deserving of a nomination for the New Year Honours list (http://www.dailymail.co.uk/news/article-5222091/Heroes-Grenfell-Tower-miss-New-Year-honours.html). This occurred even though it was an invented story.

The Holiday Inn issued a statement early the following morning confirming they did NOT have any missing children at the hotel. A police statement later that afternoon (15:49PM) reiterated “we DO NOT believe there are any accompanied children in any of the hotels in Manchester because of the explosion last night.” The lady was interviewed by the BBC Newsnight program in her own home the following day, where she recounted her narrative of events^4^ and looked visibly shaken when talking about the hundreds of calls and pictures sent to her phone from worried parents. She was not the only individual to “ghost” false missing children appeals—28 separate claims of this kind that were subsequently debunked, were identified following the Manchester Arena attack. One such false message was retweeted over 15,800 times and “liked” by in excess of 7,000 users.

A second, differently oriented, instance of event ghosting following the explosion in Manchester. This involved a Facebook post claiming that there was a gunman outside Oldham hospital. The message was imbued with urgency, written in capital letters and instructing people to avoid the area:

DO NOT COME OLDHAM HOSPITAL IM CURRENTLY LOCKED INSIDE… MAN OUTSIDE WITH GUN.

This initial post was retweeted by at least 368 Twitter accounts, many of which used a screen grab of the Facebook post referred to above, presenting it as a form of proof or “evidence.” At 00:50 (May 23, 2017), Oldham Council tweeted saying they had no information that there is a gunman at the hospital. However, this did not dispel the rumor, with new messages about “the gunman” being sent for over half an hour after this rebuttal.

This episode is especially important in demonstrating how disinformation communication can have serious and consequential effects. In the aftermath of the bombing, wracked with uncertainty about what was actually happening, the messages circulating on social media about possible attackers active in the area, caused a decision to be taken to keep some ambulances and fire crews at the outer scene cordon, for their protection. This meant they were not able to get to the victims near the bomb site who were critically injured and administer first aid.

## 7 EMULSIFYING

Emulsifying involves blending two distinct sets of ingredients together to create a new concoction. In terms of disinformation communication this technique can be applied in two ways: (1) to connect a current crisis event to wider issues or previous occurrences, thereby wrapping them into a broader narrative of grievance; and (2) complexifying the overall picture, making it harder for public audiences to discern the causes and consequences of problematic and troubling events.

In the aftermath of terror attacks, there is now an almost ritualized quality to some of the official statements made. In the hours after the violence, police will issue factual statements to update the public. Political leaders also perceive a need to communicate with the public, pivoting around a set of tropes connected to the idea that “we will not give in to terror.” Looking across the four attacks, it can be observed how these kinds of political communications can become engaged in processes of emulsification.

Following the well‐rehearsed rhythms alluded to above, the Mayor of London Sadiq Khan issued a public statement after the Westminster Bridge attack:

My statement on the incident near Parliament Square this afternoon. Please visit https://news.met.police.uk for the latest information. [attached link] (March 22, 2017; 2,566 RTs, 2,653 Likes, 668 Comments)

https://twitter.com/MayorofLondon/status/844587263828901888

Several thousand people “liked” the post, but almost all of the 668 comments were highly negative, and many aggressive. After each of the series of attacks that took place in 2017, the Mayor issued similar messages. But one in particular, where he was quoted as saying terror attacks are “part and parcel of living in a major city,” triggered particular and repeated opprobrium. Notably, President Donald Trump tweeted:

At least 7 dead and 48 wounded in terror attack and Mayor of London says there is “no reason to be alarmed!” (04/06/17—62,054 RTs, 158,819 Likes, 54K Comments)

Following on from which, a series of social media campaigns were observed that sought to causally connect the terror attacks to other social problems, especially immigration policy and refugee numbers. This pattern persisted, for example, when the Grenfell Tower tragedy happened later in 2017. Several messages questioned whether this was also “part and parcel” of living in a global city, thereby linking terrorism to other failures of social policy.

It is hard to be definitive about how emulsification works at this stage based upon the data available, and it may be that it is contextually sensitive. One plausible hypothesis is that by intertwining different subjects and narratives, the “cognitive load” placed upon audience members to follow the complexities of what is actually going on is increased, such that many are just rendered confused about how to infer causes and consequences for what has happened. Alternatively, it may be that merging separate events establishes an archetype that induces people to infer that they are of a “kind,” such that a common set of causes and consequences can be imputed. This latter model involves reducing the cognitive load engaged in interpreting and understanding through processes of simplification, by stripping out layers of context and detail (Sharot, 2017).

## 8 INFILTRATING AND INCITING

Political and public discussions of disinformation over the past two years have pivoted, to a significant degree, around the involvement of actors connected to the Kremlin and Russian State. Multiple politicians, think‐tanks, and investigative journalists have documented activities deliberately intended to disrupt democratic processes and institutions (Digital, Culture, Media and Sport Select Committee, 2018; Paul & Matthews, 2016; United States Senate, 2018). While processing the data collected in relation to the four terror attacks, a number of the Kremlin backed accounts identified by previous studies were detected, seeking to amplify the impacts of the terrorist violence.

In total, 47 accounts connected with the St Petersburg based Internet Research Agency (IRA) were identified in the current dataset. Eight of these were especially active, sending at least 475 Twitter messages across the four attacks, which were reposted in excess of 153,000 times (see Table 1).

**Table 1:** Summary of Internet Research Agency active accounts by incident

Incident	No. original messages from IRA accounts	No. of reposts
Westminster	35	35,662
Manchester	293	55,581
London Bridge	140	57,322
Finsbury Park	7	4,871

Following the Manchester and London Bridge attacks, at least one IRA account was sending inflammatory messages within 15 min:

Another day, another Muslim terrorist attack. RETWEET if you think that Islam needs to be banned RIGHT NOW! Manches… (May 22, 2017, 22:22)

From an account presenting with a right‐wing, anti‐Islam stance, this one message sent after Manchester, was retweeted 3,606 times. Responding rapidly to “frame” the definition of the situation in this manner acts to subtly shape how and what some people think. There is an “early mover advantage” to be accrued from getting in at the inception of an incident to try and sow seeds of antagonism and anxiety. The Kremlin backed accounts, whose primary purpose was to communicate disinformation, were organized around a “twin‐track” strategy of infiltrating established online thought communities and then seeking to incite and inflame their emotions, rendering them more extreme.

## 9 SPOOFING

From the evidence available, it is clear that the infiltrate and incite strategy engages several more tactical techniques of disinformation. The first of these is labeled “spoofing”—appropriating Mackenzie's (2018) term for attempts to “trick” algorithms in high frequency financial trading markets, to leverage competitive advantage and profit. Albeit focused on machines rather than humans, aspects of his analysis are redolent of Goffman's (1961, 1983) detailed dissections of how people, in their co‐present encounters and interactions with each other, deceive, misrepresent, or mask aspects of their identities and/or motives.

As a technique of disinformation, spoofing steers attention to the ways operators of fake accounts construct false digital identities. They employ these to ingratiate themselves within a digital community of individuals, seemingly possessing similar identity characteristics and/or interests to the wider group. Part of how this is done involves clear analogies with what Goffman (1961) termed “identity kits,” or the props and materials people use to symbolically display and represent a particular form of social status and positioning.

**Figure 3:**
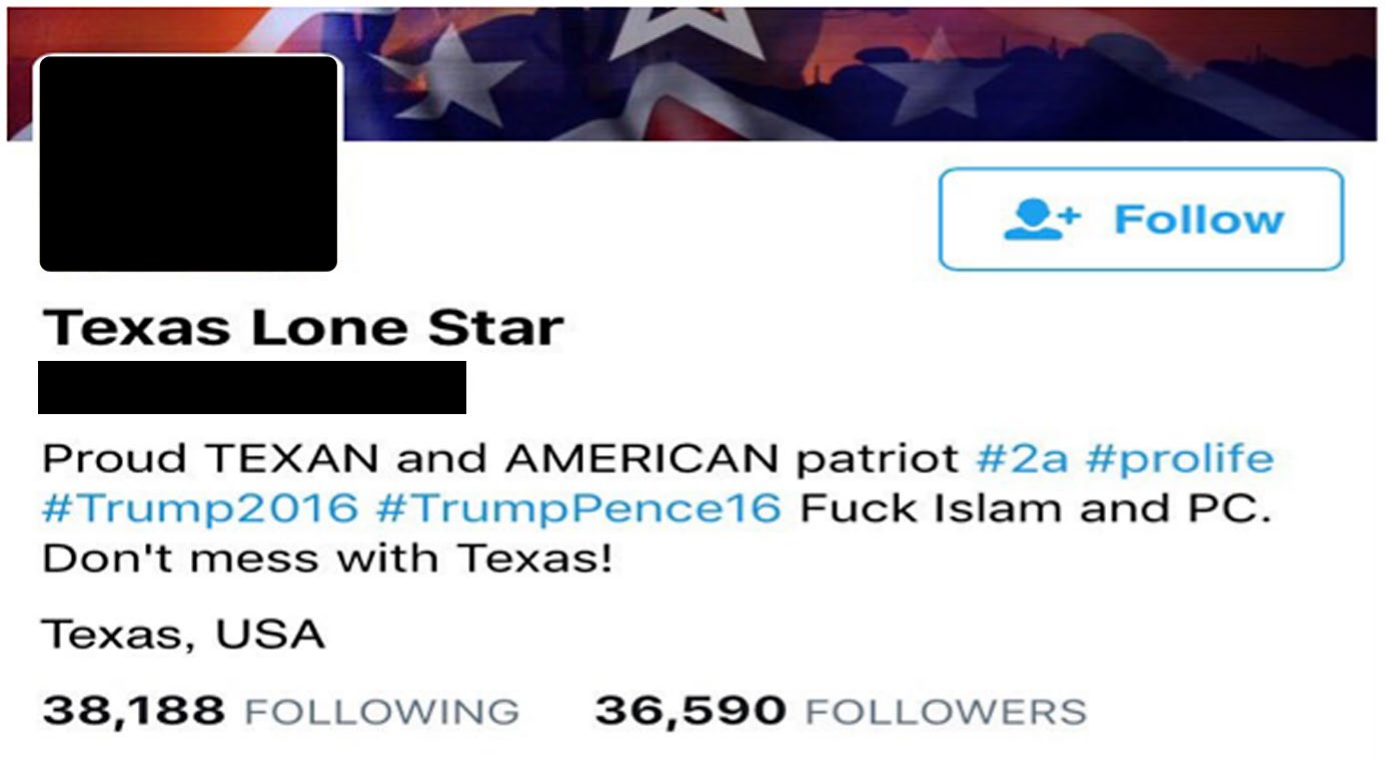
@SouthLoneStar “spoofed” Internet Research Agency profile [Colour figure can be viewed at https://www.wileyonlinelibrary.com]

For example, a number of the Kremlin backed accounts were constructed around overtly politically right‐wing, Southern state, President Trump supporting, presentations of self. The avatar pictures accompanying these accounts were stereotypical depictions of this, for example featuring white males sporting Stetson hats (see Figure 3).

@SouthLoneStar was active in messaging around a number of the episodes discussed previously. For instance, in respect of the misidentification of the suspect for the Westminster Bridge attack, the account tweeted:

UPDATE: London terrorist identified as Islamic cleric Abu Izzadeen who was sentenced to jail in January for hate p… https://t.co/Zw9uNpzB7H. RT 833 (19:01:32)

A critical feature of the infiltrate and incite strategy in general, and its use of identity spoofing as a tactic, was that the Kremlin‐backed accounts adopted a range of different personas, positioned across the ideological spectrum. This included, for example, spoofing members of the Black Lives Matter movement. The quality of mimicry and imitation was often quite convincing, allowing the operators to build up thousands of followers in some cases. This meant that around contentious and highly charged social and political issues, these accounts were simultaneously interfering in and influencing the views of multiple different digital thought communities.

## 10 “TRUTHING” AND SOCIAL PROOFING

The performance of spoofed digital identities is frequently accompanied by two other techniques of disinformation: “truthing” and “social proofing.” Owing to limitations on space and because these have been subject to a more detailed treatment in a separate article, they will only be briefly reprised here (see Innes, Dobreva, & Innes, 2019). Several of the techniques of disinformation outlined above have used visual images to try and persuade their audiences about the ultimate “truth” of their knowledge claims. Photographs and videos possess an almost inherent persuasive potency, albeit there is increasing awareness of how these too can be manipulated and “faked.” Another form of “truthing” involves presenting an argument in highly detailed and technical language, that deliberately imitates the language and representational devices of “digital forensics” and crime investigation. A third variant concerns the illegitimate manipulation of statistical data.

Assertions of “truthfulness” is also a routine feature of conspiratorial claims. When conspiracy theories are communicated they are regularly accompanied by phrases claiming that they are surfacing the “hidden” real truth about what has really occurred. What is striking about a number of the case studies considered above, and especially those involving Kremlin‐backed actors, is how there can be multiple narratives about what has happened in circulation simultaneously. Each using different materials and interpretations to buttress their apparent validity and reliability.

One additional technique of disinformation used to bolster support for soft facts harnesses some of the technological affordances designed into social media platforms. In their study of political mobilization, Margetts, John, Hale, and Yassera (2016) evidenced how the number of followers and likes attached to particular messages and accounts, influences how others interact with those materials. This can be labeled “social proofing,” in that it seeks to exploit a cognitive bias in terms of individual attention being shaped by the actions of other members of a social group (Cialdini, 2009), what Centola (2018) dubs “social reinforcement.” Extending this logic, Margetts et al.'s analysis implies that social media is better at coordinating and channeling people's opinions, than changing them.

Creating an illusion of social support for a viewpoint or idea, in order that this might persuade others to take it on, provides some insight into how and why “bots” can be deployed as part of a disinformation campaign. As automated forms of algorithmically driven communication, bots can artificially amplify the visibility of a message in the expectation that increased exposure will cause more people to align with it. Albeit, the empirical validity of such a supposition is contested (Benkler et al., 2018).

## 11 CONCLUSION: 360° DISINFORMATION

This article has distilled a series of methods via which deliberately misleading and inaccurate information is constructed and communicated to influence people's thoughts, feelings, and behavior. Some of these techniques of disinformation are engaged in “fact softening”; disrupting and undermining audience beliefs in the veracity of the information. Counter‐pointed with these are other techniques that “harden” less empirically supported interpretations. That multiple techniques of disinformation can be detected, sometimes overlapping and other times in tension with each other, gestures towards the complex nature of disinformation communication as a public policy problem. Indeed, it is notable that as part of attempting to understand how disinformation functions, this analysis has encompassed the full spectrum of media from peer‐to‐peer platforms, through mainstream mass media outlets, “bots” and “trolling” behavior.

In identifying these communication tactics and techniques, the intent has been to derive a set of insights that are conceptually driven, and thus agnostic about the specific socio‐technical affordances associated with individual social media platforms. They are more generalizable and transferable. The analogy with Sykes and Matza's (1957) framing is instructive in that where they were studying deviant behavior, herein the discussion has pivoted around “deviant” information.

Matching the descriptive analytic tone that colored Sykes and Matza's original approach, a key feature of the techniques of disinformation is that they move beyond any misplaced assumption that such forms of communicative action are only engaged by hostile states as part of their geopolitical machinations. Instead, they start to key us into how the performance of disinforming communications is becoming a more commonplace feature of the ordering of social reality in the information age.

The experience of disinformation for many citizens, in many countries, is that it does not reach them via one vector, but rather swirls around them in multiple forms, pervading and permeating their political and digital lives. It is more of a “360 degree” encounter.

That said, while we can highlight that disinformation and other modes of soft fact are increasingly profligate and distributed throughout the contemporary media ecosystem, less certain is “how much this actually matters?” So while the causes and consequences of disinformation is being constructed as a public problem eliciting considerable concern, several notable studies have cautioned that it should not be over‐inflated in terms of its “real‐world” social and political impacts (Benkler et al., 2018).

There is increasing research evidence of how particular disinformation strategies and tactics have been operationalized to influence and subvert the integrity of democratic processes and systems. This article, has sought to understand how soft facts have been communicated in the wake of terrorist events and the impacts induced, reflecting how the immediate period following a crisis event is frequently highly uncertain and contingent. As such, people are rendered susceptible to digital  influence engineering techniques, and thus disinformation communicated during these moments can prove profoundly influential upon definitions of the situation and processes of social reaction.

## Data Availability

Data available on request due to privacy/ethical restrictions.

## NOTES

1 An excellent summary can be found at: https://www.nytimes.com/interactive/2018/09/20/us/politics/russia-interference-election-trump-clinton.html (accessed September 21, 2018).

2 See https://democrats-intelligence.house.gov/social-media-content/ (accessed October 2, 2018).

3 For an overview of memes and their functions see Shifman (2013).

4 In this narrative, the individual talks about two children and shouting out to other children, with no mention of the “50 children” in her message that she said she would “look after.”
